# Differential Effects of Short-Chain Fatty Acids on L6 Myotube Inflammatory Mediator Production in Response to Lipopolysaccharide- or Palmitic Acid-Stimulation

**DOI:** 10.3390/nu14142826

**Published:** 2022-07-09

**Authors:** Jamie L. A. Martin, Nadia M. Cartwright, Amber L. Hutchinson, Lindsay E. Robinson, David W. L. Ma, Jennifer M. Monk

**Affiliations:** Department of Human Health and Nutritional Sciences, University of Guelph, Guelph, ON N1G 2W1, Canada; jmarti35@uoguelph.ca (J.L.A.M.); nadiacartwright.4@gmail.com (N.M.C.); hutchina@uoguelph.ca (A.L.H.); lrobinso@uoguelph.ca (L.E.R.); davidma@uoguelph.ca (D.W.L.M.)

**Keywords:** L6 myotubes, skeletal muscle, short-chain fatty acids, inflammatory mediators, lipopolysaccharide, palmitic acid, cytokines, chemokines, butyrate

## Abstract

Short-chain fatty acids (SCFA) produced from dietary non-digestible carbohydrate fermentation have metabolic effects in skeletal muscle; however, their effect on inflammatory mediator production is unknown. In this study, L6 myotubes were cultured with individual SCFA (acetate, propionate, and butyrate) at 0.5 mM and 2.5 mM ± 10 ng/mL lipopolysaccharide (LPS) or ± 500 µM palmitic acid (PA) for 24 h. In response to LPS, only butyrate had an effect at the lower concentration (0.5 mM), whereas at the higher concentration (2.5 mM) both propionate and butyrate reduced MCP-1, MIP-1α, and RANTES secretion (*p* < 0.05), and only butyrate reduced IL-6 secretion and intracellular protein levels of phospho-STAT3 (*p* < 0.05). In response to PA, 0.5 mM butyrate reduced protein expression of phospho-NFκB p65 and the secretion of IL-6, MIP-1α, and MCP-1, whereas all three SCFA reduced RANTES secretion (*p* < 0.05). At the 2.5 mM SCFA concentration combined with PA stimulation, all three SCFA reduced intracellular protein expression of phospho-NFκB p65 and phospho-STAT3 and secreted protein levels of MCP-1, IL-6, and RANTES, whereas only butyrate reduced secretion of MIP-1α (*p* < 0.05). Thus, SCFA exhibit differential effects on inflammatory mediator expression in response to LPS and PA stimulation, which has implications for their individual impacts on inflammation-mediated skeletal muscle dysfunction.

## 1. Introduction

Short-chain fatty acids (SCFA) are produced from the anaerobic bacterial fermentation of non-digestible carbohydrates (NDC) by the microbiota in the gastrointestinal tract [1,2,3]. In the colon, SCFA (acetate, propionate, and butyrate), are typically produced in the molar ratio of 3:1:1, respectively, having a combined concentration that has been shown to range between 20 and 150 mM [4,5,6]. However, fecal and circulating SCFA levels are variable based on dietary intakes of different amounts and types of NDC (which are not equally fermentable), the composition of the microbiota, and abundance of SCFA-producing microbial species [7,8,9,10]. Subsequently, SCFA can enter the portal circulation wherein mean plasma levels are significantly lower and variable compared to fecal levels [11,12,13,14,15,16]. Once in the circulation, SCFA may impact host physiological function and have been studied for their effects on whole body energy homeostasis and metabolism, with tissue specific effects documented in the liver, adipose tissue, pancreas, and skeletal muscle, as reviewed elsewhere [2,17,18,19].

SCFA have been shown to impact skeletal muscle metabolic function, thereby highlighting the potential impact of microbial-derived bioactives like SCFA to alter host physiological function given that skeletal muscle contributes to 30% of resting energy expenditure [20] and 80% of insulin-stimulated glucose uptake [21]. Oral injection of acetate (5.2 mg/kg, 5 days/week over 6 months) administered to insulin-resistant obese rats improved glucose tolerance and reduced body weight, liver lipogenesis, and lipid accumulation in adipose tissue [22]. Using the same model, acetate increased abdominal skeletal muscle mRNA expression of glucose transporter type 4 (GLUT4) and myoglobin [23]. Similar results were observed in L6 myotube cells stimulated with 0.5 mM acetate, resulting in increased insulin-stimulated glucose uptake and protein expression of GLUT4, myoglobin, and the transcription factor promoting their expression, myocyte enhancer 2A (MEF2A) [24,25]. These results are indicative of acetate increasing glucose uptake, an effect observed elsewhere for both acetate and propionate [26]; however, this outcome was not shown with butyrate [27]. Interestingly, butyrate did improve insulin signaling in an established palmitic acid (PA)-induced insulin resistant state [27]. As well, sodium butyrate supplementation (5% *w/w*) to a high fat diet in mice attenuated obesity-associated insulin resistance by reducing fasting blood glucose and insulin levels and homeostasis model assessment-estimated insulin resistance (HOMA-IR) [24,28]. Moreover, in the same study, skeletal muscle from butyrate-supplemented obese mice exhibited a change in muscle morphology evidenced by increased type-1 fiber content, fat oxidation, and expression of phosphorylated-AMP-activated protein kinase (p-AMPK) and peroxisome proliferator-activated receptor γ coactivator-1α (PGC-1α) [24,28]. In vitro studies using the L6 myotube cells have also shown similar outcomes with acetate [25] and butyrate [24,28], resulting in increased fatty acid oxidation and expression of p-AMPK and PGC-1α. Moreover, 0.5 mM acetate enhanced fatty acid metabolism, evidenced by increased protein expression of phosphorylated acetyl-CoA carboxylase (p-ACC), increased fatty acid uptake, and reduced PA-stimulated triglyceride accumulation in L6 myotubes [24]. Similar findings of decreased skeletal muscle lipid accumulation have been shown in animal models that were supplemented with butyrate or acetate [29,30,31].

Despite what is known about the metabolic effects of some SCFA types (predominantly acetate) in skeletal muscle [23,24,26,27,28,29,30,31], little is known about their effects on inflammatory mediator production (e.g., tumor necrosis factor (TNF)α, interleukin (IL)-6, monocyte chemoattractant protein (MCP)-1, etc.), which is important to characterize since pro-inflammatory mediator production can adversely impact skeletal muscle function in obesity, as reviewed elsewhere [32]. Inflammatory chemokines, such as MCP-1, regulated on activation, normal T cell expressed, and secreted (RANTES), and macrophage inflammatory protein (MIP)-1α, are also involved in the recruitment of immune cells to obese and insulin-resistant skeletal muscle [33,34]. MCP-1 secretion has been observed to be elevated in insulin-resistant human skeletal muscle cells [34], and RANTES protein expression, which is mediated by nuclear factor kappa light chain enhancer of activated B cells (NFκB) activation [35], has been shown to be upregulated by as much as 80% in the skeletal muscle of obese rats [33]. MIP-1α expression in skeletal muscle along with other cytokines and chemokines further contribute to pro-inflammatory crosstalk between immune cells and skeletal muscle, as reviewed elsewhere [36]. In other cell types, SCFA have been shown to exert some anti-inflammatory effects via modulation of NFκB activation in immune cells [33]. Similarly, vascular smooth muscle cells treated with 5 mM butyrate for 24 h were shown to increase both total and phosphorylated NFκB p65 protein expression [34].

The objective was to examine the modulation of L6-myotube-derived inflammatory mediator production by different types of SCFA (acetate, propionate, and butyrate) at low and high concentrations in the absence or presence of a lipopolysaccharide (LPS)-induced inflammatory microenvironment designed to mimic the obese state, or a PA-induced insulin-resistant state.

## 2. Materials and Methods

### 2.1. Cell Culture and Differentiation

L6 myoblasts were purchased from American Type Culture Collection (ATCC; CRL-1458; Manassas, VA, USA) and were grown and passaged according to the manufacturer’s specifications. Cells were maintained in a humidified incubator at 5% carbon dioxide and 37 °C, in Dulbecco’s modified Eagle’s medium (DMEM) containing 4 mmol/L L-glutamine, 4500 mg/L glucose, and 1 mM sodium pyruvate (HyClone, Logan, UT, USA), which was supplemented with 10% (*v/v*) low endotoxin sterile-filtered fetal bovine serum (FBS; Millipore-Sigma, Oakville, ON, Canada) and 1% (*v/v*) penicillin-streptomycin (HyClone) [26,27,35]. Myoblasts (at passage 6) were seeded in 6-well plates (Fisher Scientific, Mississauga, ON, Canada) at 4 × 10^4^ cells/mL, and upon reaching 70–80% confluence, the cells were differentiated and maintained as multi-nucleated myotubes with striated fibers in DMEM supplemented with 2% (*v/v*) FBS and 1% (*v*/*v*) penicillin-streptomycin. Media were changed on days 2, 4, and 6, and on day 7, the media were replaced with serum-reduced DMEM containing 0.25% (*v*/*v*) FBS and 1% (*v*/*v*) penicillin-streptomycin for 12 h prior to experiments on day 8.

### 2.2. L6 Myotube Experimental Conditions

L6 myotube cultures were treated with either media alone as a control (Con) or with one of three individual SCFA; namely, (i) sodium acetate (Ace), (ii) sodium propionate (Pro), and (iii) sodium butyrate (But) (each from Millipore-Sigma) at a final concentration of either 0.5 mM (low concentration) or 2.5 mM (high concentration) in serum-reduced DMEM for 24 h, and each treatment condition was either unstimulated (i.e., cells ± SCFA alone) or stimulated with LPS or PA. Thus, each of the aforementioned SCFA treatment conditions was stimulated with either 10 ng/mL of LPS from *Escherichia coli* 055:B5 (Millipore-Sigma), which recapitulates the circulating endotoxin levels reported in obese humans [36] and rodent high-fat diet-induced obesity models [37,38], or 500 µM of palmitic acid (PA, ≥ 98% pure, Caymen Chemicals, Ann Arbor, MI, USA) to induce insulin resistance, as shown previously [39,40]. A stock solution of PA was prepared using lab-grade ethanol and complexed to 2% (*w*/*v*) bovine serum albumin (BSA; containing ≤0.1 ng/mg endotoxin and ≤0.02% fatty acids; Millipore-Sigma) dissolved in serum-reduced DMEM pre-warmed to 37 °C prior to its addition to the L6 myotube cultures.

### 2.3. RNA Isolation and PCR Analysis

RNA and protein were isolated from each cell culture treatment condition using the RNA/Protein Purification Plus Kit (Norgen Biotek Corp., Thorold, ON, Canada) per the manufacturer’s instructions. Subsequently, 2 μg of cDNA was synthesized using a high-capacity cDNA reverse transcription kit (Applied Biosystems, Foster City, CA, USA). Real-Time PCR was performed using a CFX Real-Time PCR System (Bio-Rad, Mississauga, ON, Canada) as previously described [41]. Primers were designed using the Universal Probe Library Assay Design Center (Roche Applied Sciences, Penzberg, Germany), and validated primer efficiencies were between 90% and 105%. Primer sequences are shown in Table 1. Results were normalized to the housekeeping gene β-actin and relative differences in gene expression (expressed in arbitrary units) between treatment groups were determined according to the calculation 2^(40-Ct)^.

### 2.4. Intracellular and Secreted Protein Analysis

Total intracellular protein was quantified using the bicinchoninic assay according to the manufacturer’s instructions (Fisher Scientific). An equal amount of protein (10 μg/sample) was utilized to simultaneously measure phosphorylated-NFκB p65 (Ser536) and phosphorylated-signal transducer and activator of transcription 3 (STAT3) (Ser727) using the Bio-Plex Pro Cell Signaling kit (Bio-Rad) per the manufacturer’s instructions. Secreted cytokines (IL-6) and chemokines (MCP-1, MIP-1α, MIP-1β, and RANTES) were measured in culture supernatant using the Bio-Plex Pro Cytokine 6-plex kit as per the manufacturer’s instructions. Intracellular and secreted protein endpoints were analyzed using the Bio-Plex 200 system/Bio-Plex Manager software, Version 6.0 (Bio-Rad). Secreted TNFα protein in the culture supernatant was measured using the rat TNFα ELISA kit as per the manufacturer’s instructions (Invitrogen, Burlington, ON, Canada) and the optical densities for all samples were above the detection limit of the assay.

### 2.5. Statistical Analysis

All data are expressed as mean  ±  SEM. Gene expression data were analyzed by two-way ANOVA (main effects: SCFA treatment and stimulus), and intracellular and secreted protein expression within either LPS or PA-stimulated groups were analyzed by one-way ANOVA (main effect: SCFA treatment). All ANOVA analyses were followed by Tukey’s multiple comparison test for post hoc analyses between groups (*p* < 0.05). The Shapiro–Wilk test was used to test for normality. Analyses were conducted using GraphPad Prism 9.3.0 (GraphPad Software, Inc., La Jolla, CA, USA).

## 3. Results

### 3.1. Effect of SCFA on Inflammatory Mediator Expression and Secretion in LPS-Stimulated L6 Myotubes

**Figure 1:**
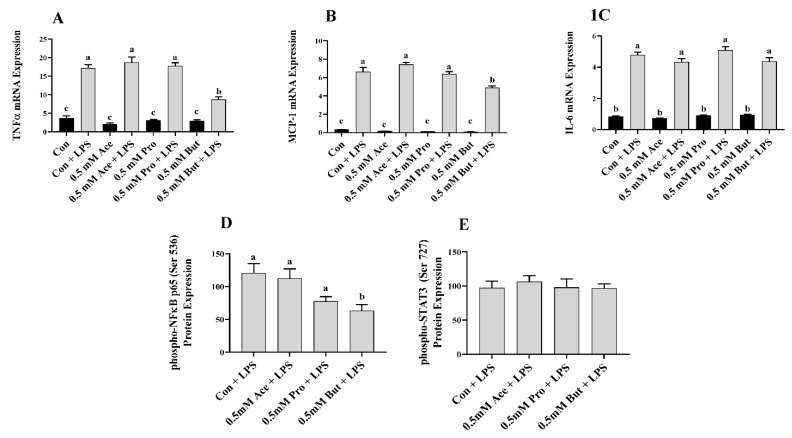
Effect of 0.5 mM SCFA ± LPS stimulation on L6 myotube inflammatory mediator mRNA expression (**A**–**C**) and LPS-stimulated phosphorylated transcription factor intracellular protein levels (**D**,**E**). Values are means ± SEM (*n* = 8/group). Data were analyzed by two-way ANOVA (**A**–**C**), or one-way ANOVA (**D**,**E**) followed by Tukey’s multiple comparison test. Bars not sharing a lower-case letter differ (*p* < 0.05). SCFA treatment without LPS (black bars) and SCFA with LPS (grey bars).

Initially, L6 myotubes were stimulated with LPS at a dose that recapitulates the circulating level in obese humans and rodents [36,37,38]. The effect of a low dose of individual SCFA on inflammatory mediator mRNA expression in response to LPS is shown in Figure 1. There was no difference in the level of inflammatory mediator mRNA expression between Con and any SCFA in the unstimulated (i.e., no LPS) treatment condition (*p* > 0.05; Figure 1A–C). Further, LPS stimulation increased mRNA expression of all inflammatory mediators in comparison to unstimulated in all treatment groups (*p* < 0.05; Figure 1A–C). Within the LPS-stimulated L6 myotube cultures, only But reduced the level of mRNA expression of *Tnfα* and *Mcp-1* compared to Con (*p* < 0.05), whereas Ace and Pro did not differ from Con (Figure 1A,B). Conversely, there was no difference in the level of *Il-6* mRNA expression in response to LPS stimulation between any SCFA and Con (*p* > 0.05, Figure 1C). Intracellular protein levels of the phosphorylated form of the transcription factors that drive inflammatory mediator production exhibited a similar outcome and are shown in Figure 1D,E. Under LPS-stimulated conditions, only But reduced the level of L6 myotube phospho-NFκB p65 intracellular protein levels compared to Con (*p* < 0.05), whereas the expression level in Ace- and Pro-treated cells did not differ from Con (*p* > 0.05, Figure 1D).

**Figure 2:**
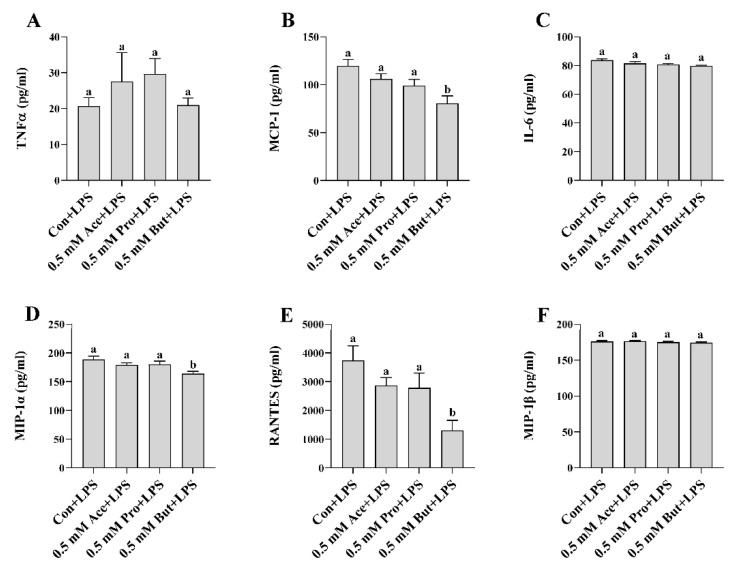
Effect of 0.5 mM SCFA + LPS stimulation on L6 myotube secreted protein levels of inflammatory mediators (**A**–**F**). Values are means ± SEM (*n* = 8/group). Data were analyzed by one-way ANOVA followed by Tukey’s multiple comparison test. Bars not sharing a lower-case letter differ (*p* < 0.05).

Similar to the outcome for *Il-6* mRNA expression, the level of phospho-STAT3 intracellular protein did not differ between any SCFA and Con (*p* > 0.05, Figure 1E). Secreted cytokines and chemokines in the culture supernatant in response to LPS stimulation are shown in Figure 2A–F. Only But reduced the secreted levels of the chemokines MCP-1, MIP-1α, and RANTES compared to Con (*p* < 0.05, Figure 2B,D,E). There was no difference in secreted protein levels of TNFα, IL-6, and MIP-1β between treatment groups (*p* > 0.05. Figure 2A,C,F).

**Figure 3:**
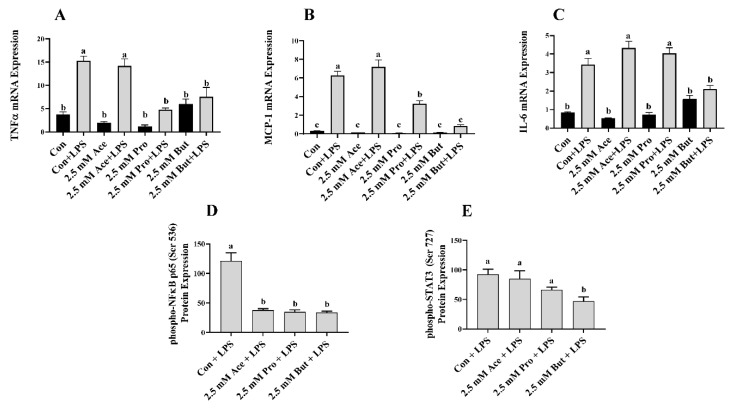
Effect of 2.5 mM SCFA ± LPS stimulation on L6 myotube inflammatory mediator mRNA expression (**A**–**C**) and LPS-stimulated phosphorylated transcription factor intracellular protein level (**D**,**E**). Values are means ± SEM (*n* = 8/group). Data were analyzed by two-way ANOVA (**A**–**C**), or one-way ANOVA (**D**,**E**) followed by Tukey’s multiple comparison test. Bars not sharing a lower-case letter differ (*p* < 0.05). SCFA treatment without LPS (black bars) and SCFA with LPS (grey bars).

**Figure 4:**
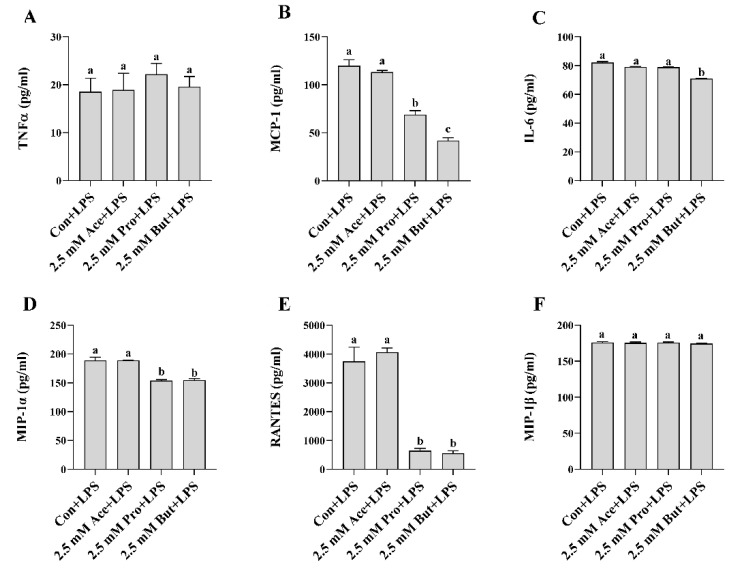
Effect of 2.5 mM SCFA + LPS stimulation on L6 myotube secreted protein level of inflammatory mediators (**A–F**). Values are means ± SEM (*n* = 8/group). Data were analyzed by one-way ANOVA followed by Tukey’s multiple comparison test. Bars not sharing a lower-case letter differ (*p* < 0.05).

Next, we assessed inflammatory mediator mRNA and activated (i.e., phosphorylated) transcription factor protein expression in LPS-stimulated L6 myotube cultures treated with a higher concentration (2.5 mM) of each SCFA (Figure 3). There was no difference in inflammatory mediator mRNA expression between Con and any SCFA in the unstimulated cultures (*p* > 0.05). As expected, LPS stimulation increased inflammatory mediator mRNA expression compared to unstimulated conditions (*p* < 0.05; Figure 3A–C), with the exception of *Tnfα* mRNA expression in Pro- and But-treated cultures (*p* > 0.05; Figure 3A) and *Mcp-1* and *Il-6* mRNA expression in But-treated cultures ((*p* > 0.05; Figure 3B,C), which did not differ between unstimulated and LPS-stimulated treatments. Within LPS-stimulated cultures, both Pro and But reduced the level of *Tnfα* mRNA expression compared to Con (*p* < 0.05, Figure 3A). Conversely, the level of *Tnfα* mRNA expression in Ace + LPS-treated cultures did not differ from Con + LPS (*p* > 0.05). A similar expression pattern was observed for *Mcp-1*, wherein both Pro and But LPS-stimulated cultures exhibited reduced mRNA expression levels compared to Con + LPS (*p* < 0.05, Figure 3B); however, there was no difference in *Mcp-1* mRNA expression levels between Ace- and Con-treated cultures in response to LPS (*p* > 0.05). *Il-6* mRNA expression was only reduced in But + LPS-treated cultures compared to Con + LPS (*p* < 0.05, Figure 3C), whereas the expression level in Ace- and Pro-treated cultures did not differ from Con in response to LSP (*p* > 0.05). LPS-stimulated intracellular protein levels of phospho-NFκB p65 were reduced by all three SCFA compared to Con (*p* < 0.05, Figure 3D), whereas only But reduced phospho-STAT3 protein expression compared to Con (*p* < 0.05, Figure 3E). LPS-stimulated secreted protein levels of cytokines and chemokines are shown in Figure 4. Only But reduced IL-6 secreted protein levels compared to Con (*p* < 0.05, Figure 4C). Additionally, MCP-1, MIP-1α, and RANTES secreted protein levels were reduced by both Pro and But in comparison to Con, whereas there was no difference between Ace and Con (*p* < 0.05, Figure 4B,D,E). There was no difference between treatment groups in secreted protein levels of TNFα and MIP-1β (*p* > 0.05, Figure 4A,F). Collectively, these results demonstrate the ability of SCFA to reduce inflammatory cytokine and chemokine protein secretion in response to LPS stimulation in L6 myotubes.

### 3.2. Effect of SCFA on Inflammatory Mediator Expression and Secretion in PA-Stimulated L6 Myotubes

**Figure 5:**
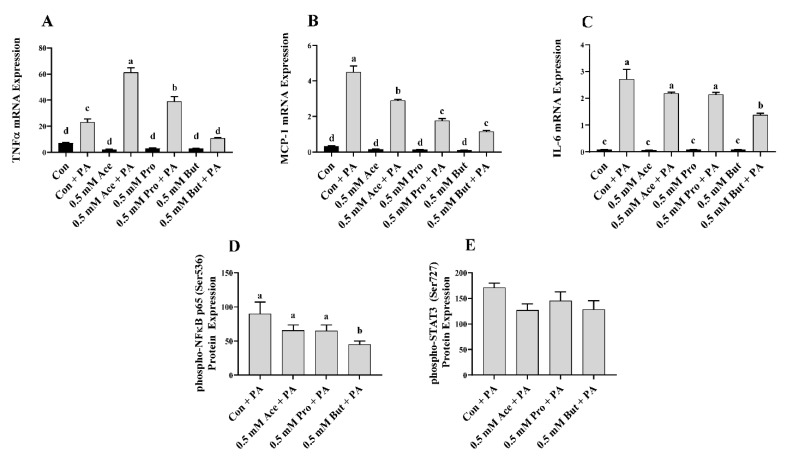
Effect of 0.5 mM SCFA ± PA stimulation on L6 myotube inflammatory mediator mRNA expression (**A**–**C**) and PA-stimulated phosphorylated transcription factor intracellular protein level (**D**,**E**). Values are means ± SEM (*n* = 8/group). Data were analyzed by two-way ANOVA (**A**–**C**), or one-way ANOVA (**D**,**E**) followed by Tukey’s multiple comparison test. Bars not sharing a lower-case letter differ (*p* < 0.05). SCFA treatment without PA (black bars) and SCFA with PA (grey bars).

SCFA treated L6 myotubes were also stimulated with PA to induce insulin resistance, as demonstrated previously [39,40]. As expected, the level of inflammatory mediator mRNA expression in response to PA was increased compared to the unstimulated condition (culture media alone with and without SCFA), as shown in Figure 5A–C, with the exception of *Tnfα* mRNA expression, which did not differ between But and But + PA cultures (*p* > 0.05; Figure 5A). At the lower concentration of SCFA (0.5 mM) stimulated with PA, both Ace and Pro increased *Tnfα* mRNA expression levels compared to Con, whereas But decreased TNFα mRNA expression compared to Con (*p* < 0.05, Figure 5A). *Mcp-1* mRNA expression levels were decreased by all three SCFA compared to Con, wherein expression was lower for Pro and But compared to Ace (*p* < 0.05, Figure 5B). Finally, only But reduced IL-6 mRNA expression levels compared to Con (*p* < 0.05, Figure 5C), whereas there was no difference between Ace, Pro, and Con (*p* > 0.05).

**Figure 6:**
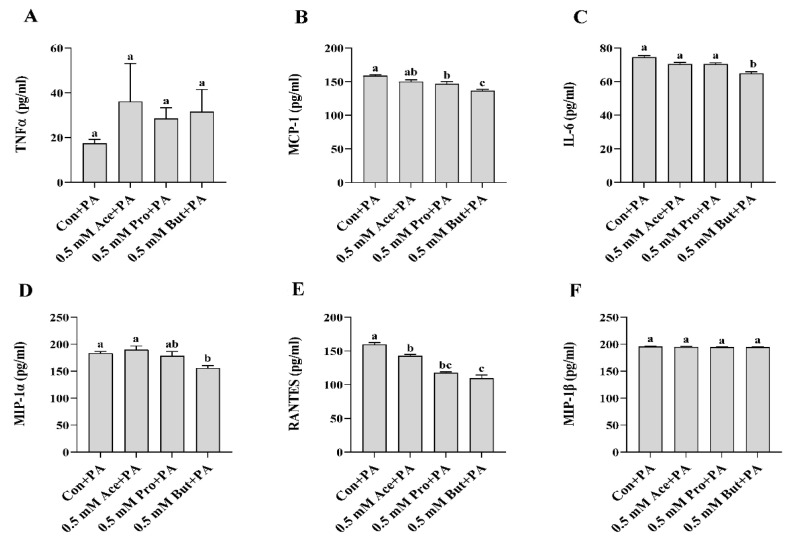
Effect of 0.5 mM SCFA + PA stimulation on L6 myotube secreted protein level of inflammatory mediators (**A**–**F**). Values are means ± SEM (*n* = 8/group). Data were analyzed by one-way ANOVA followed by Tukey’s multiple comparison test. Bars not sharing a lower-case letter differ (*p* < 0.05).

With respect to inflammatory transcription factor intracellular protein levels, only But reduced the level of phospho-NFκB p65 compared to Con (*p* < 0.05, Figure 5D), whereas Ace and Pro did not differ from Con (*p* > 0.05). There was no difference in the level of phospho-STAT3 protein expression between any treatment groups in response to PA stimulation (*p* > 0.05, Figure 5E). Cytokine and chemokine secreted protein levels in response to PA stimulation are shown in Figure 6. There was no difference in secreted TNFα between groups (*p* > 0.05, Figure 6A), whereas only But reduced IL-6 secreted protein levels compared to Con (*p* < 0.05, Figure 6C). Both Pro and But decreased MCP-1 secretion compared to Con (*p* < 0.05, Figure 6B), whereas there was no difference between Con and Ace (*p* > 0.05). Only But reduced MIP-1α secretion compared to Con (*p* < 0.05, Figure 6D), whereas there was no difference between Ace and Pro compared to Con (*p* > 0.05). Conversely, all three SCFA reduced the secreted protein levels of RANTES compared to Con, wherein But had the lowest levels (*p* < 0.05, Figure 6E). There was no difference in secreted MIP-1β levels between treatment groups (*p* > 0.05, Figure 6F).

**Figure 7:**
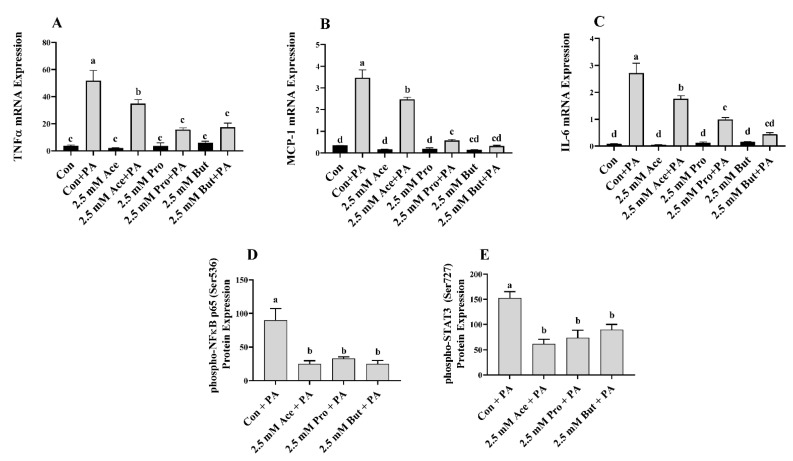
Effect of 2.5 mM SCFA ± PA stimulation on L6 myotube inflammatory mediator mRNA expression (**A**–**C**) and PA-stimulated phosphorylated transcription factor intracellular protein level (**D**,**E**). Values are means ± SEM (*n* = 8/group). Data were analyzed by two-way ANOVA (**A**–**C**), or one-way ANOVA (**D**,**E**) followed by Tukey’s multiple comparison test. Bars not sharing a lower-case letter differ (*p* < 0.05). SCFA treatment without PA (black bars) and SCFA with PA (grey bars).

**Figure 8:**
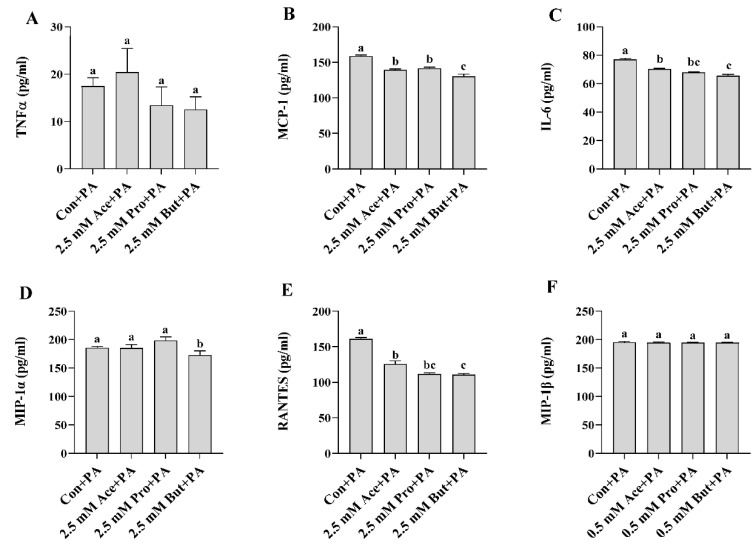
Effect of 2.5 mM SCFA + PA stimulation on L6 myotube secreted protein levels of inflammatory mediators (**A**–**F**). Values are means ± SEM (*n* = 8/group). Data were analyzed by one-way ANOVA followed by Tukey’s multiple comparison test. Bars not sharing a lower-case letter differ (*p* < 0.05).

At the higher concentration of SCFA (2.5 mM), there was no difference in inflammatory mediator mRNA expression between Con and any SCFA in the unstimulated treatment condition (*p* > 0.05; Figure 7A–C). PA stimulation increased inflammatory mediator mRNA expression compared to the unstimulated condition for all groups (*p* < 0.05), with the exception of *Tnfα* mRNA expression in Pro- and But-treated cultures (Figure 7A) and *Mcp-1* and *Il-6* mRNA expression in But-treated cultures (Figure 7B,C). Within PA-treated cultures, all three SCFA reduced the level of *Tnfα* mRNA expression compared to Con (*p* < 0.05, Figure 7A). Ace exhibited an intermediate effect, and the *Tnfα* mRNA expression level was lowest in Pro- and But-treated L6 myotube cultures. A similar expression profile was apparent for both *Mcp-1* and *Il-6* expression in response to PA stimulation, wherein all three SCFA reduced the mRNA expression level of both mediators compared to Con with the lowest expression in Pro- and But-treated cultures (*p* < 0.05, Figure 7B,C). In response to PA, intracellular protein levels of both phospho-NFκB p65 and phospho-STAT3 were reduced for each SCFA compared to Con, but there was no difference between Ace, Pro, and But (*p* < 0.05, Figure 7D,E). Secreted protein levels in PA-stimulated cultures are shown in Figure 8, wherein there was no difference between treatment groups in TNFα or MIP-1β levels (*p* > 0.05, Figure 8A,F). All three SCFA reduced IL-6 secreted protein levels compared to Con (*p* > 0.05, Figure 8C), wherein But-treated cultures had the lowest levels and Ace and Pro exhibited intermediate levels. Similarly, all three SCFA (Ace, Pro, and But) reduced MCP-1 secretion compared to Con (*p* > 0.05, Figure 8C), wherein But had the lowest levels that were significantly reduced compared to Ace and Pro. Only But reduced MIP-1α secreted protein levels compared to Con (*p* < 0.05, Figure 8D), whereas there was no difference between Ace or Pro and Con (*p* > 0.05). All three SCFA (Ace, Pro, and But) reduced RANTES secreted protein levels compared to Con (*p* > 0.05, Figure 8E), wherein levels were the lowest in But-treated cultures compared to Con, and Ace and Pro exhibited an intermediate effect. Collectively, these results demonstrate the ability of SCFA to reduce inflammatory cytokine and chemokine protein secretion in response to PA stimulation in L6 myotubes.

## 4. Discussion

The current study demonstrated the effects of individual SCFA (Ace, Pro, and But) on L6 myotube inflammatory mediator production in response to either LPS or PA stimulation. These effects were tested at high and low molar concentrations that have been used previously in cell culture studies utilizing L6 myotubes [24,25]. Importantly, these concentrations reflect the range of SCFA concentrations observed in human blood [11,12,13,14,15,16], which can be highly variable and highlight the need for testing the effects of each SCFA at lower concentrations. Despite the demonstrated anti-inflammatory effects of SCFA (or chemical agonists for SCFA signaling receptors) on immune cells [33,42] and vascular smooth muscle cells [34], the effect of SCFA on skeletal muscle cell inflammatory mediator production is unknown. This is in contrast to the metabolic effects of SCFA on skeletal muscle function, which has been reviewed elsewhere [19], which include increasing insulin-stimulated glucose uptake in L6 myotubes and/or improving skeletal muscle glucose tolerance in vivo [22,24,25,26,27,28] and an acetate-specific effect of increasing fatty acid oxidation [24,28]. Importantly, the acetate molar concentration (0.5 mM) that was observed to induce metabolic improvements in L6 myotubes [24,25] was also used in the current study, thereby serving as a more physiologically relevant SCFA concentration that better reflects the variable circulating concentration levels [11,12,13,14,15,16]. The higher SCFA molar concentration of 2.5 mM used in the current study is central within the range of SCFA molar concentrations (1–5 mM) that have been used previously to demonstrate beneficial effects on cardiomyocyte function [43] and in vascular smooth muscle cells [25,34,44,45,46,47]. The dose of LPS used to stimulate an inflammatory cellular microenvironment recapitulates the circulating endotoxin levels reported in obese humans and rodents [36,37,38]. Additionally, the dose of PA used in the current study has been shown to induce skeletal muscle cell insulin resistance while maintaining cell viability [39,40]. Both LPS and PA are ligands that signal through toll-like receptor-4 (TLR4), a receptor for which expression is increased in obese skeletal muscle and downstream signaling results in inflammatory mediator secretion and insulin resistance, as shown in L6 myotubes and skeletal muscle [48,49]. Therefore, the use of these two stimulation conditions in L6 myotube cell cultures reproduces critical aspects of the obese skeletal muscle microenvironment in vitro and provides a model to discern the acute effects of individual SCFA on local inflammatory mediators (i.e., myokines), which when produced locally exert autocrine or paracrine effects on myocyte metabolic function [32].

TLR4 signaling leads to the activation of the NFκB pathway [48,49], which results in the secretion of inflammatory mediators (e.g., TNFα, MCP-1, and IL-6) and stimulates insulin resistance [32,50,51]. Mechanistically, NFκB induces serine phosphorylation of insulin receptor substrate 1 (IRS-1), which impairs insulin-induced tyrosine phosphorylation, and thus, inhibits downstream insulin signaling [52]. STAT3 activation (phosphorylation at serine 727) has been shown to occur downstream of TLR4 signaling and contributes to the secretion of IL-6 and MCP-1 [53]. Moreover, non-canonical STAT3 activation is required for TLR4-mediated metabolic and inflammatory effects [53]. In this connection, palmitic acid (as palmitate) treatment of L6 myotubes increased STAT3 phosphorylation and induced insulin resistance, which was prevented by silencing STAT3 [54]. Secretion of TNFα, IL-6, and MCP-1 from both myocytes and skeletal muscle are increased in chronic conditions such as obesity and have been shown to contribute to impaired glucose uptake and insulin resistance [32,51]. Thus, the impact of SCFA on inflammatory transcription factors (NFκB p65 and STAT3) and/or inflammatory mediators including cytokines (TNFα and IL-6) and chemokines (MCP-1, MIP-1α, MIP-1β, and RANTES) has the potential to not only reduce local inflammation, but also beneficially impact skeletal muscle metabolic function.

In the current study, individual SCFA exhibited different effects on L6 myotube expression and/or secretion of inflammatory mediators in response to either LPS or PA stimulation that was dependent on their molar concentration and type/carbon number (i.e., Ace, Pro, or But). In response to LPS at the lower SCFA dose (0.5 mM; Figure 1 and Figure 2), only But reduced (i) intracellular protein levels of phospho-NFκB p65, (ii) secretion of MIP-1α and RANTES, and (iii) both mRNA expression and secreted protein levels of MCP-1. At the higher SCFA concentration (2.5 mM; Figure 3 and Figure 4), only Pro and But reduced the level of secreted MCP-1, MIP-1α and RANTES protein, whereas all three SCFA reduced the intracellular protein level of phospho-NFκB p65. Interestingly, only But reduced both *Il-6* mRNA expression and IL-6 secreted protein levels along with the intracellular protein level of the corresponding transcription factor phospho-STAT3. Despite differences in the gene expression of TNFα in response to different SCFA, the observed changes in mRNA expression did not translate to secreted protein levels in response to LPS, which were not significantly different between SCFA. Overall secretion of TNFα was very low versus that of other secreted cytokines and chemokines; however, the detected levels of TNFα were above the detection limit of the assay. Additionally, despite reduced expression of phospho-NFκB p65 intracellular protein expression in Ace-treated cells after 24 h LPS stimulation, gene expression and secreted protein levels of downstream inflammatory mediators, such as TNFα and MCP-1, did not differ from control. This may reflect that the effects of different SCFA on the kinetics of inflammatory mediator expression and/or secretion are not equivalent and further study is required.

In response to PA at the lower SCFA dose (Figure 5 and Figure 6), the reduction in inflammatory mediator secretion followed a pattern of But >Pro >Ace. Specifically, But exerted the most potent effect by reducing activation (i.e., phosphorylation) of NFκB p65 intracellular protein and the secretion of MCP-1, MIP-1α, and RANTES. Despite a reduction in *Tnfα* mRNA expression by But and an increase in *Tnfα* mRNA expression by Ace and Pro (Figure 5A), these changes did not translate to the secreted protein levels, and further study is required to investigate the effect of different SCFA on the kinetics of the TNFα response to PA stimulation in L6 myotubes. In response to PA stimulation, Pro exhibited a more intermediate effect, reducing secreted protein levels of MCP-1 and RANTES, and only Ace reduced RANTES secretion. At the higher dose of SCFA (Figure 7 and Figure 8), the effects of SCFA were more pronounced, wherein all three SCFA reduced intracellular protein levels of phospho-NFκB p65 and phospho-STAT3, which drive the expression of downstream inflammatory mediators. All SCFA reduced RANTES protein secretion, whereas only But reduced MIP-1α secretion. Gene expression levels aligned with secreted protein levels of IL-6 and MCP-1, which were reduced by all SCFA; however, this was not the case for TNFα expression, which may reflect a differential kinetic response for this cytokine within a 24 h period and further study is required to assess the kinetic response of all cytokines and chemokines in response to stimulation in this model. Additionally, further mechanistic and functional studies are required to discern the implications of SCFA reducing skeletal muscle cell inflammatory cytokine and chemokine levels. Thus, possible future directions include identifying the mechanisms through which individual SCFA modify TLR-mediated signaling and/or insulin signaling pathways, metabolic function (e.g., insulin-stimulated glucose uptake), and the functional implications of reduced immune cell (e.g., monocyte), chemotaxis, and macrophage polarization, and the subsequent effect on myocyte-immune cell paracrine signaling.

The data from the current study underscore that the effects of different SCFA are not physiologically equivalent. L6 myotubes exhibit different patterns of inflammatory mediator mRNA expression and/or secreted protein and intracellular transcription factor activation in response to different SCFA concentrations combined with different physiologically relevant stimulation conditions (i.e., LPS and PA). Although frequently considered to function similarly, SCFA of varying carbon number (i.e., Ace, Pro, and But) are biologically unique molecules that have been shown to exhibit varying binding affinities for different signaling receptors (including G protein-coupled receptors (GPR)-41, -43, and 109a, and the aryl hydrocarbon receptor) and the inhibition of histone deacetylases, as reviewed elsewhere [33,55]. Moreover, the tissue distribution of different SCFA signaling receptors is variable [33], resulting in unique tissue-specific signaling mechanisms that underlie the physiological effects of each individual SCFA, which require further study in skeletal muscle and other tissue sites. This is akin to the variability in SCFA-mediated effects on intestinal epithelial barrier function [56,57,58,59,60,61,62,63]. Additionally, further study is required to assess both metabolic and inflammatory function of individual and combined SCFA at physiologically relevant molar concentrations to comprehensively understand the effects of individual SCFA on skeletal muscle function, as the current study provides the first evidence of unique inflammatory suppressive effects of different SCFA in response to LPS or PA stimulation. Gastrointestinal SCFA production is variable and confounded by factors that will influence the molar concentration and profile of SCFA in the circulation that can reach skeletal muscle and other peripheral tissues. These confounding factors include variability in the (i) dietary intake of the amount and type(s) of NDC (namely SCFA precursors, e.g., soluble fibers, resistant starches, and oligosaccharides), (ii) the relative fermentability of NDC by the microbiota (i.e., fast versus slow), (iii) the microbiota composition of SCFA-producing microbial species, and (iv) host intestinal absorptive capacity [7,8,9,10]. Identifying the biological effects of individual SCFA at physiologically relevant concentrations to demonstrate their therapeutic potential to impact skeletal muscle function and local inflammatory mediator production will better inform dietary guidelines for NDC intakes. This approach will help to ensure that optimal concentrations of the microbial-derived bioactive metabolites required to impact host physiological function will be produced in vivo from dietary precursors to support skeletal muscle function.

## Figures and Tables

**Table 1:** Primer sequences.

Gene	Forward Primer (5′-3′)	Reverse Primer (5′-3′)
*β-actin*	accgagcatggctacagcgtcacc	gtggccatctcttgctcggagtct
*Il-6*	cccttcaggaacagctatgaa	acaacatcagtcccaagaagg
*Mcp-1*	cgtgctgtctcagccagat	ggatcatcttgccagtgaatg
*Tnfα*	gcccagaccctcacactc	ccactccagctgctcctct

## Data Availability

Data are contained within the article. The data presented in this study are available upon request to the corresponding author.
